# Marital status and survival in patients with non-small cell lung cancer: an analysis of 70006 patients in the SEER database

**DOI:** 10.18632/oncotarget.21568

**Published:** 2017-10-06

**Authors:** Ying Wu, Zisheng Ai, Guotong Xu

**Affiliations:** ^1^ Postdoctoral Research Station of Medicine, Tongji University School of Medicine, Shanghai, China; ^2^ Department of Medical Statistics, Tongji University School of Medicine, Shanghai, China; ^3^ Tongji Eye Institute, Tongji University School of Medicine, Shanghai, China

**Keywords:** non-small cell lung cancer, marital status, cancer survival, SEER

## Abstract

Marital status has been demonstrated to be related to the survival of patients in various cancer types, but the relationship in the large population of non-small cell lung cancer (NSCLC) has rarely been studied. In this study, we retrospectively extracted 70006 eligible NSCLC patients from the Surveillance, Epidemiology, and End Results (SEER) database in the period from 2004 to 2012. Marital status was categorized as married, divorced/separated, widowed, and never married. Chi-square tests were used to investigate the association between marital status and other variables. The Kaplan-Meier test was adopted to compare survival curves of different groups. Multivariate Cox regression analyses were conducted to estimate the effect of marital status on overall survival (OS) and NSCLC cause-specific survival (CSS). We further performed subgroup analyses according to TNM stage and surgery condition. The results showed that marital status was an independent prognostic factor for OS and CSS of NSCLC patients. Subgroup analyses showed that the relationship between marital status and prognosis varies according to different conditions. Widowed patients with surgery were at greater risk of death across all stages and non-surgical unmarried patients at advanced stages suffered poorer prognosis than the married. To conclude, in the NSCLC patients, married patients had advantage over the unmarried in both OS and CSS.

## INTRODUCTION

Lung cancer is the most common cancer among men worldwide in terms of incidence and mortality, and among women has the third highest incidence and is second after breast cancer in mortality [1]. American Cancer Society has estimated that there will be 222,500 new cases and 155,870 deaths caused by lung cancer in United States in 2017 [2]. Although the development of newer, advanced treatment has improved the outcomes of patients, lung cancer remains the leading cause of cancer-related mortality in the United States. The 5-year relative survival rate from 1995 to 2001 for lung cancer patients were only 15.7%, much lower than other cancers [3].

Non-small cell lung cancer (NSCLC) is any type of epithelial lung cancer other than small cell lung cancer, and accounts for about 85% of all lung cancer cases [4]. A variety of clinicopathologic factors have been identified of the prognostic importance, such as the presence of pulmonary symptoms, non-squamous histology, larger tumor size, vascular invasion, etc. The influence of many demographic factors such as age and sex on the prognosis have also been investigated. During the year 2006-2008, Kumi et al. conducted researches on the relationship between marital status and survival in NSCLC patients in Japan [5, 6]; and Jatoi et al. investigated into the impact of marital status on NSCLC patients’ survival and quality of life [7]. The three studies found no evidence supporting marital status was associated with survival in all NSCLC patients. However, in recent years, many researches have demonstrated that marital status independently predicts the survival of a series of cancers including gastric cancer [8–10], colorectal cancer [11, 12], liver cancer [13], pancreatic cancer [14], and several other types of cancer [15–18], with larger population from the US Surveillance, Epidemiology and End Results (SEER) cancer database.

Therefore, in this study, we aim to perform a comprehensive population-based analysis using SEER database to clarify the prognosis significance of marital status on the survival of NSCLC patients. We used data from 2004 to 2012 to investigate the risk of overall survival and cancer-specific survival associated with marital status and further analyzed the risk according to different stages.

## RESULTS

### Patient baseline characteristics

A total of 70006 eligible NSCLC patients were identified during the study period (from 2004 to 2012) in the SEER database, including 37074(52.96%) male and 32932(47.04%) female patients. Among them, 37209(53.15%) were married, 10108(14.44%) were divorced or separated, 13251(18.93%) were widowed, and 9438(13.48%) were never married. Table 1 represents the summary of the subgroups of each variable and the relationship between each variable and marital status. Significant differences were observed in all subgroups. Specifically, married group had the highest proportion of male patients (62.34%), while widowed group had the highest proportion of female patients (73.29%). Widowed patients were more likely to be over 80 years (40.92%), while most of the never married patients were less than 60 4142(43.89%). White patients accounted for the majority of each marital group, but the proportion of black patients were higher in never married group (28.63%) than that in other groups. Never married patients were more likely to be stage IV (50.94%), and married patients were more likely to receive surgery (29.29%), compared with other groups.

**Table 1:** Baseline characteristics of NSCLC patients

Characteristics	Total N (%)	Married N (%)	Divorced/ Separated N (%)	Widowed N (%)	Never married N (%)	*P* value
	70006(100)	37209(53.15)	10108(14.44)	13251(18.93)	9438(13.48)	
Gender						<0.0001
Male	37074(52.96)	23197(62.34)	4936(48.83)	3540(26.71)	5401(57.23)	
Female	32932(47.04)	14012(37.66)	5172(51.17)	9711(73.29)	4037(42.77)	
Age						<0.0001
<60	16297(23.28)	8498(22.84)	3086(30.53)	571(4.31)	4142(43.89)	
60-69	20697(29.56)	12073(32.45)	3711(36.71)	2101(15.86)	2812(29.79)	
70-79	21087(30.12)	11609(31.20)	2571(25.44)	5157(38.92)	1750(18.54)	
≥80	11925(17.03)	5029(13.52)	740(7.32)	5422(40.92)	734(7.78)	
Race						<0.0001
White	54400(77.71)	29838(80.19)	7809(77.26)	10689(80.67)	6064(64.25)	
Black	8695(12.42)	2884(7.75)	1717(16.99)	1392(10.50)	2702(28.63)	
Others	6911(9.87)	4487(12.06)	582(5.76)	1170(8.83)	672(7.12)	
Diagnosis year						<0.0001
2004-2008	37086(52.98)	19825(53.28)	5179(51.24)	7230(54.56)	4852(51.41)	
2009-2012	32920(47.02)	17384(46.72)	4929(48.76)	6021(45.44)	4586(48.59)	
Median household income						<0.0001
Quartile 4	17719(25.31)	9167 (24.64)	2667 (26.39)	3239 (24.44)	2646 (28.04)	
Quartile 3	18935(27.05)	10154(27.29)	2833 (28.03)	3592 (27.11)	2356 (24.96)	
Quartile 2	16263(23.23)	8766 (23.56)	2497 (24.70)	3209 (24.22)	1791 (18.98)	
Quartile 1	17089(24.41)	9122 (24.52)	2111 (20.88)	3211 (24.23)	2645 (28.03)	
Grade						<0.0001
I	3870(5.53)	2253(6.05)	440(4.35)	773(5.83)	404(4.28)	
II	12649(18.07)	7042(18.93)	1809(17.90)	2221(16.76)	1577(16.71)	
III	18919(27.02)	10332(27.77)	2753(27.24)	3228(24.36)	2606(27.61)	
IV	1345(1.92)	746(2.00)	197(1.95)	229(1.73)	173(1.83)	
Unknown	33223(47.46)	16836(45.25)	4909(48.57)	6800(51.32)	4678(49.57)	
TNM stage						<0.0001
I	16037(22.91)	8681(23.33)	2204(21.80)	3380(25.51)	1772(18.78)	
II	3678(5.25)	2070(5.56)	527(5.21)	609(4.60)	472(5.00)	
III	18166(25.95)	9510(25.56)	2659(26.31)	3611(27.25)	2386(25.28)	
IV	32125(45.89)	16948(45.55)	4718(46.68)	5651(42.65)	4808(50.94)	
Histology						<0.0001
adenocarcinoma	32981(47.11)	18370(49.37)	4620(45.71)	5592(42.20)	4399(46.61)	
squamous carcinoma	15892(22.70)	8271(22.23)	2451(24.25)	3072(23.18)	2098(22.23)	
Others	21133(30.19)	10568(28.40)	3037(30.05)	4587(34.62)	2941(31.16)	
Surgery						<0.0001
Yes	18372(26.24)	10900(29.29)	2608(25.80)	2740(20.68)	2124(22.50)	
No	51634(73.76)	26309(70.71)	7500(74.20)	10511(79.32)	7314(77.50)	
Radiotherapy						<0.0001
Yes	31144(44.49)	16940(45.53)	4781(47.30)	4976(37.55)	4447(47.12)	
No	38862(55.51)	20269(54.47)	5327(52.70)	8275(62.45)	4991(52.88)	

### Effect of marital status on overall and cause-specific survival

**Figure 1:**
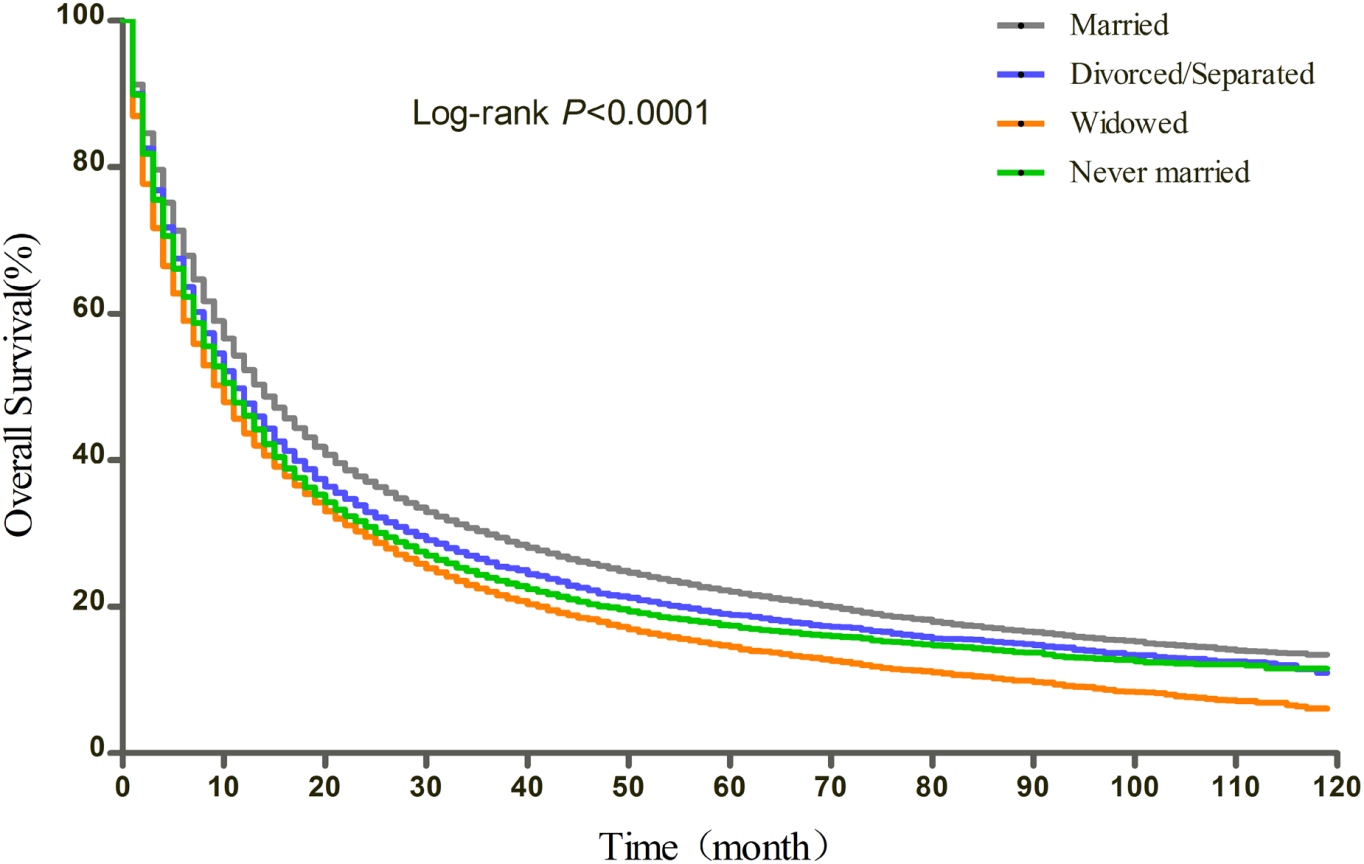
Kaplan-Meier curves of the effect of marital status on overall survival (OS)

**Figure 2:**
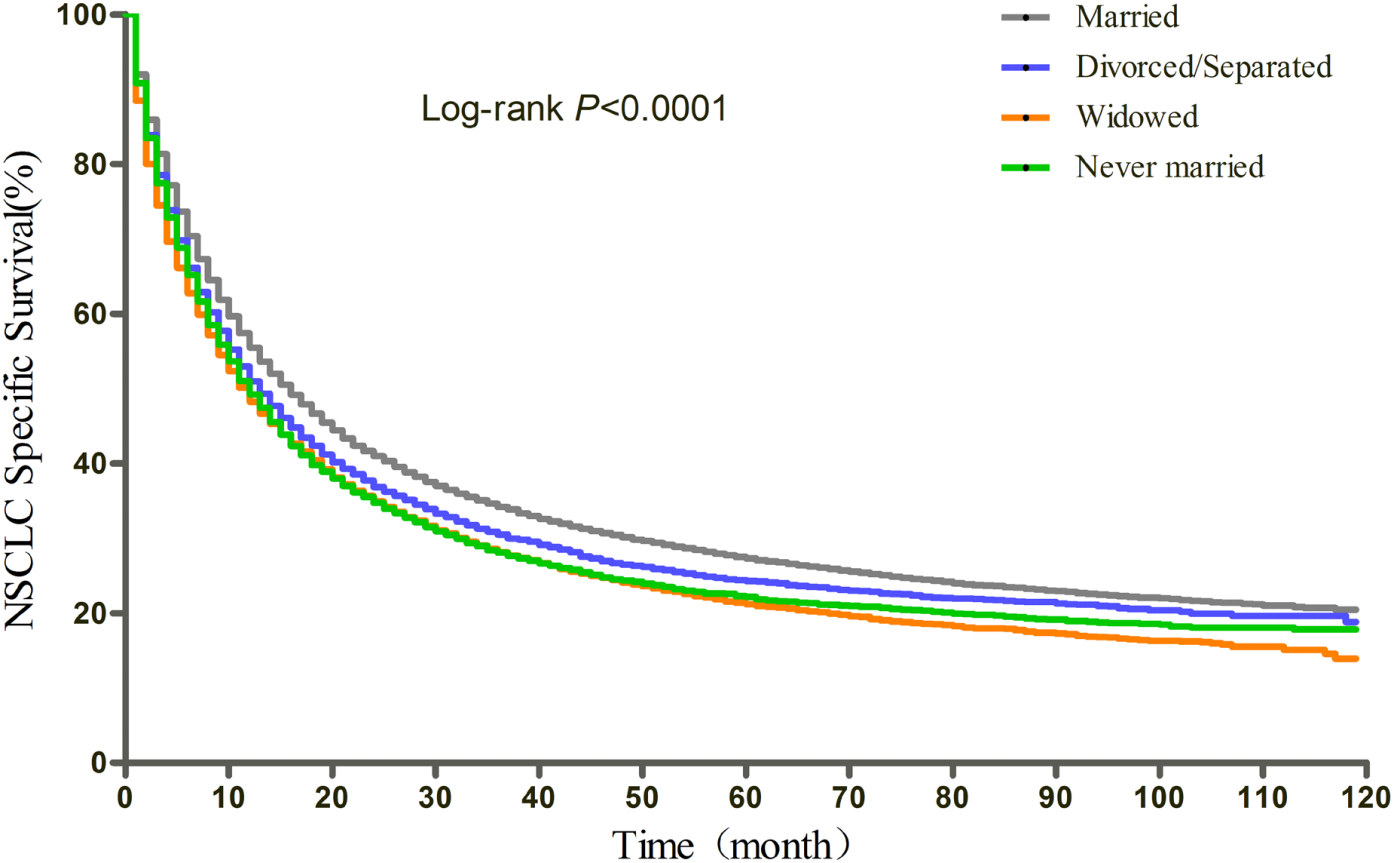
Kaplan-Meier curves of the effect of marital status on NSCLC cause-specific survival (CSS)

The results of Kaplan-Meier tests and multivariate Cox analysis of the effect of marital status and covariates on OS and CSS were shown in Table 2 and Table 3, respectively. The median OS was 14 months for the married, 11 months for the divorced/separated and the never married, and 10 months for the widowed (log-rank test p<0.0001) (Figure 1). After adjusting for other factors with Cox regression, marital status was found to be an independent prognostic factor of OS. Divorced/separated (HR=1.15, 95%CI: 1.12-1.18), widowed (HR=1.16, 95%CI: 1.14-1.19), and never married (HR=1.15, 95%CI: 1.12-1.18) patients had an increased risk of mortality compared with married patients. In terms of CSS, the median CSS was 16 months for married patients, 13 months for divorced/separated patients, 12 months for widowed and never married patients (log-rank test p<0.0001) (Figure 2). Similarly, after adjusting all covariates, marital status was still identified as significantly associated with the CSS. Divorced/separated (HR=1.14, 95%CI: 1.11-1.17), widowed (HR=1.15, 95%CI: 1.12-1.18), and never married (HR=1.13, 95%CI: 1.10-1.16) patients had an increased risk of NSCLC cause-specific mortality compared with married patients. Besides, female was associated with better OS and CSS and other races was a protective factor for NSCLC compared with white patients. However, age over 60, higher and unknown grade, higher TNM stage, squamous carcinoma and other histological types, lower median household income (Quartile 1 and 2 compared with Quartile 4), no surgery, and no radiotherapy were identified as risk factors of both OS and CSS.

**Table 2:** Univariate and multivariate analyses of overall survival (OS)

Characteristics	Median OS(month)	Univariate analysis		Multivariate analysis	
		Log-rank	*P*	HR(95%CI)	*P*
Marital status		544.62	<.0001		
Married	14			Ref.	
Divorced/separated	11			1.15(1.12, 1.18)	<.0001
Widowed	10			1.16(1.14, 1.19)	<.0001
Never married	11			1.15(1.12, 1.18)	<.0001
Gender		530.73	<.0001		
Male	11			Ref.	
Female	14			0.81(0.80, 0.83)	<.0001
Age		1486.10	<.0001		
<60	14			Ref.	
60-69	14			1.13(1.11, 1.16)	<.0001
70-79	12			1.34(1.30, 1.37)	<.0001
≥80	8			1.65(1.61, 1.70)	<.0001
Race		131.47	<.0001		
White	12			Ref.	
Black	10			1.01(0.98, 1.03)	0.5546
Others	14			0.87(0.84, 0.89)	<.0001
Diagnosis year		53.38	<.0001		
2004-2008	12			Ref.	
2009-2012	13			0.93(0.91, 0.95)	<.0001
Median household income		116.53	<.0001		
Quartile 4	13			Ref.	
Quartile 3	13			0.99(0.97, 1.02)	0.6257
Quartile 2	12			1.05(1.03, 1.08)	<.0001
Quartile 1	11			1.05(1.02, 1.07)	0.0003
Grade		8018.64	<.0001		
I	64			Ref.	
II	32			1.35(1.28, 1.42)	<.0001
III	13			1.55(1.47, 1.63)	<.0001
IV	10			1.66(1.54, 1.79)	<.0001
Unknown	8			1.49(1.42, 1.57)	<.0001
TNM stage		21640.45	<.0001		
I	63			Ref.	
II	30			1.60(1.53, 1.67)	<.0001
III	13			1.85(1.79, 1.90)	<.0001
IV	6			3.23(3.13, 3.33)	<.0001
Histology		2490.73	<.0001		
adenocarcinoma	16			Ref.	
squamous carcinoma	13			1.14(1.11, 1.16)	<.0001
Others	8			1.15(1.13, 1.18)	<.0001
Surgery		19418.35	<.0001		
Yes	71			Ref.	
No	8			2.67(2.59, 2.76)	<.0001
Radiotherapy		1563.04	<.0001		
Yes	10			Ref.	
No	15			1.14(1.12, 1.16)	<.0001

**Table 3:** Univariate and multivariate analyses of NSCLC cause-specific survival (CSS)

Characteristics	Median CSS (month)	Univariate analysis		Multivariate analysis	
		Log-rank	*P*	HR(95%CI)	*P*
Marital status		338.87	<.0001		
Married	16			Ref.	
Divorced/separated	13			1.14(1.11, 1.17)	<.0001
Widowed	12			1.15(1.12, 1.18)	<.0001
Never married	12			1.13(1.10, 1.16)	<.0001
Gender		408.42	<.0001		
Male	12			Ref.	
Female	17			0.83(0.81, 0.84)	<.0001
Age		707.69	<.0001		
<60	15			Ref.	
60-69	16			1.10(1.07, 1.13)	<.0001
70-79	14			1.25(1.22, 1.28)	<.0001
≥80	9			1.51(1.46, 1.55)	<.0001
Race		115.70	<.0001		
White	14			Ref.	
Black	12			0.99(0.96, 1.02)	0.3895
Others	17			0.85(0.82, 0.88)	<.0001
Diagnosis year		51.66	<.0001		
2004-2008	13			Ref.	
2009-2012	15			0.93(0.91, 0.94)	<.0001
Median household income		96.96	<.0001		
Quartile 4	15			Ref.	
Quartile 3	15			0.99(0.96, 1.02)	0.5201
Quartile 2	14			1.05(1.02, 1.07)	0.0009
Quartile 1	12			1.04(1.01, 1.07)	0.0032
Grade		7661.50	<.0001		
I	72.23^*^			Ref.	
II	43			1.42(1.34, 1.51)	<.0001
III	15			1.65(1.56, 1.75)	<.0001
IV	11			1.78(1.63, 1.93)	<.0001
Unknown	9			1.57(1.48, 1.66)	<.0001
TNM stage		23688.82	<.0001		
I	81.40^*^			Ref.	
II	38			1.96(1.86, 2.06)	<.0001
III	15			2.39(2.30, 2.47)	<.0001
IV	6			4.37(4.22, 4.53)	<.0001
Histology		2048.99	<.0001		
adenocarcinoma	19			Ref.	
squamous carcinoma	15			1.11(1.08, 1.13)	<.0001
Others	9			1.12(1.10, 1.15)	<.0001
Surgery		18624.76	<.0001		
Yes	80.20^*^			Ref.	
No	9			2.74(2.65, 2.84)	<.0001
Radiotherapy		1787.39	<.0001		
Yes	11			Ref.	
No	19			1.11(1.09, 1.13)	<.0001

^*^ represents the mean survival month since the median survival month is not available.

### Subgroup analyses of patients with surgery stratified by TNM stage

**Figure 3:**
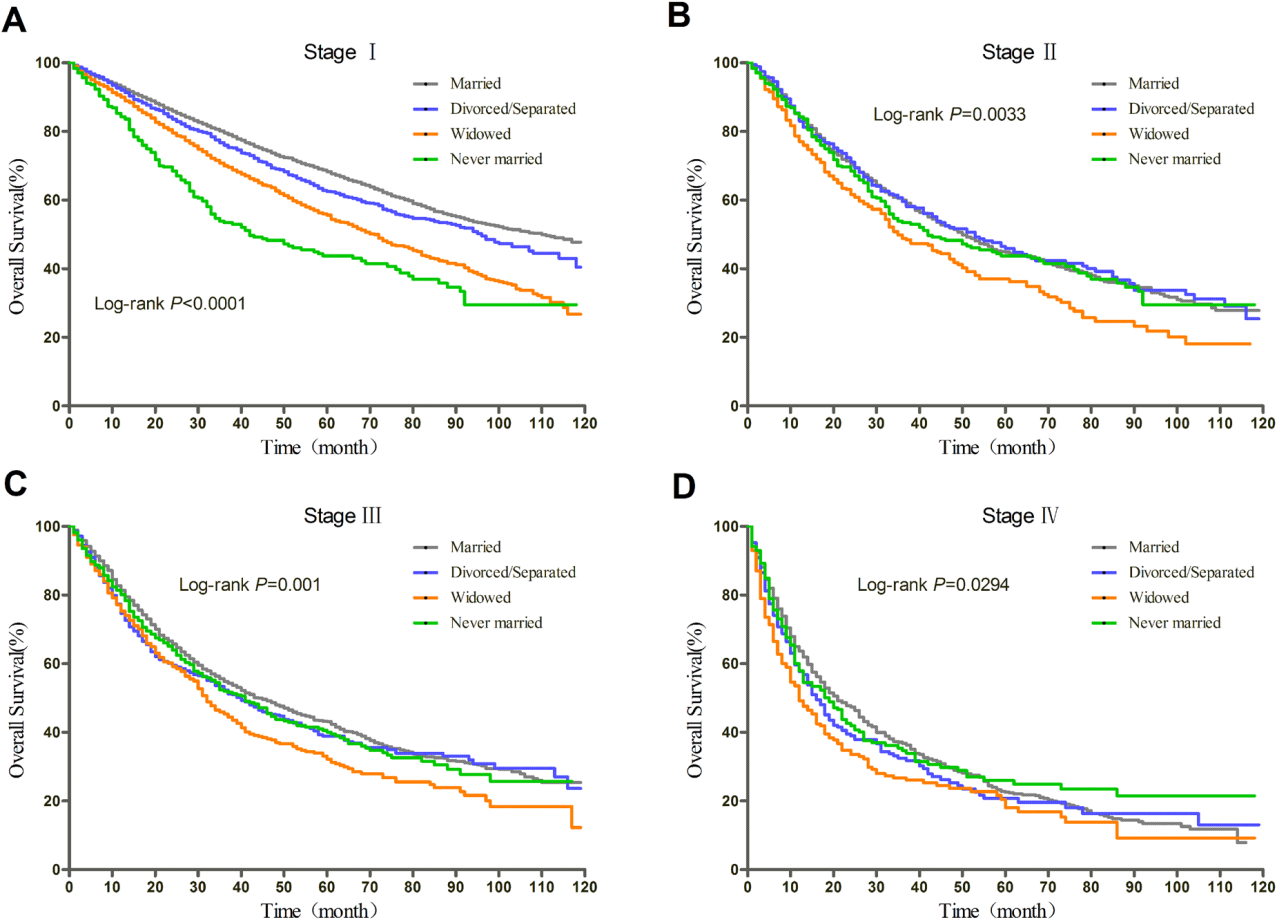
Kaplan-Meier curves of the effect of marital status on OS for patients with surgery by stage

**Figure 4:**
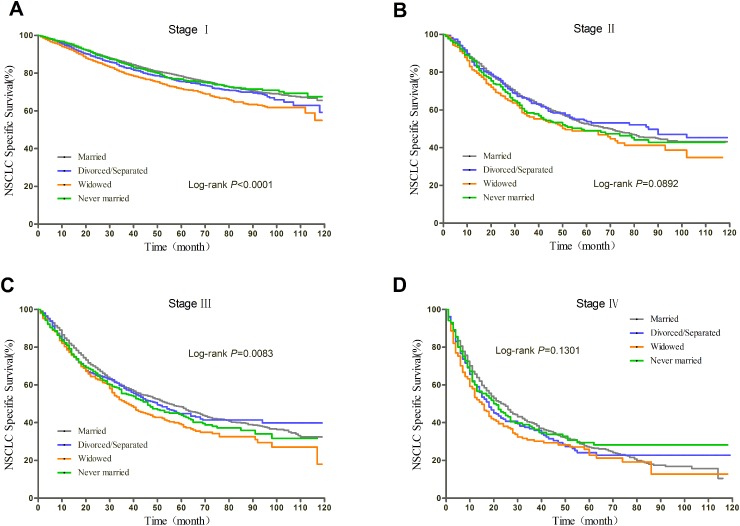
Kaplan-Meier curves of the effect of marital status on CSS for patients with surgery by stage

Prognosis of NSCLC varies much according to TNM stage and surgery condition. Therefore, we further explored the effect of marital status on OS and CSS, stratified by TNM, in patients who received surgery. The log-rank tests of the OS and CSS differences among different marital status were shown in Figure 3 and Figure 4, respectively. The results were summarized in Table 4 for OS and Table 5 for CSS. After adjusting other covariates in Cox regression, divorced/separated, widowed, and never married had greater risk of overall mortality compared with married patients at Stage I and Stage III. Widowed patients and married patients had poorer prognosis compared with married patients at Stage II, and only widowed patients showed poorer prognosis at Stage IV. In the context of CSS, compared with married patients, divorced/separated showed greater hazard ratio of mortality at Stage I, widowed patients had poorer prognosis at Stage I, III and IV, and never married patients had higher risk of mortality at Stage II and III. Besides, female patients had better OS and CSS than male patients at all stages. Age over 70 was risk factor at every stage. For Stage I, II, and IV patients with surgery, not receiving radiotherapy was identified as a protective factor for OS and CSS.

**Table 4:** Subgroup analyses stratified by TNM stage for NSCLC patients with surgery (OS)

Characteristics	Stage I	Stage II	Stage III	Stage IV
Marital status				
Married	Ref.	Ref.	Ref.	Ref.
Divorced/separated	1.31(1.19, 1.44)^a^	1.11(0.94, 1.32)	1.20(1.06, 1.37)^a^	1.14(0.95, 1.37)
Widowed	1.36(1.24, 1.48)^a^	1.23(1.04, 1.47)^b^	1.25(1.08, 1.44)^a^	1.36(1.12, 1.66)^a^
Never married	1.17(1.04, 1.31)^a^	1.24(1.04, 1.47)^b^	1.19(1.03, 1.38)^b^	0.94(0.77, 1.16)
Gender				
Male	Ref.	Ref.	Ref.	Ref.
Female	0.67(0.63, 0.72)^a^	0.76(0.68, 0.86)^a^	0.76(0.69, 0.84)^a^	0.76(0.67, 0.87)^a^
Age				
<60	Ref.	Ref.	Ref.	Ref.
60-69	1.48(1.33, 1.65)^a^	1.16(1.00, 1.35)	1.05(0.94, 1.18)	1.09(0.93, 1.27)
70-79	2.20(1.98, 2.45)^a^	1.75(1.50, 2.05)^a^	1.47(1.30, 1.66)^a^	1.25(1.04, 1.49)^b^
≥80	3.37(2.97, 3.81)^a^	2.10(1.69, 2.61)^a^	1.85(1.54, 2.23)^a^	1.48(1.15, 1.89)^a^
Race				
White	Ref.	Ref.	Ref.	Ref.
Black	1.15(1.02, 1.29)^b^	0.83(0.67, 1.01)	0.93(0.79, 1.09)	1.14(0.94, 1.40)
Others	0.89(0.78, 1.01)	0.75(0.61, 0.92)^a^	0.90(0.76, 1.06)	0.93(0.73, 1.19)
Diagnosis year				
2004-2008	Ref.	Ref.	Ref.	Ref.
2009-2012	0.90(0.84, 0.97)^a^	0.99(0.87, 1.12)	0.88(0.80, 0.97)^a^	0.90(0.79, 1.02)
Median household income				
Quartile 4	Ref.	Ref.	Ref.	Ref.
Quartile 3	1.14(1.04, 1.26)^a^	1.01(0.86, 1.20)	1.16(1.01, 1.33)^b^	1.08(0.90, 1.29)
Quartile 2	1.14(1.05, 1.25)^a^	0.91(0.78, 1.06)	1.08(0.95, 1.22)	1.06(0.89, 1.26)
Quartile 1	1.01(0.92, 1.12)	0.89(0.76, 1.05)	0.97(0.85, 1.10)	0.93(0.77, 1.13)
Grade				
I	Ref.	Ref.	Ref.	Ref.
II	1.48(1.33, 1.65)^a^	1.07(0.82, 1.40)	1.63(1.31, 2.03)^a^	1.22(0.94, 1.59)
III	1.68(1.50, 1.88)^a^	1.24(0.95, 1.62)	1.90(1.53, 2.37)^a^	1.57(1.22, 2.04)^a^
IV	1.69(1.35, 2.12)^a^	1.15(0.77, 1.71)	2.50(1.80, 3.46)^a^	1.69(1.14, 2.51)^a^
Unknown	1.43(1.23, 1.66)^a^	1.30(0.94, 1.80)	1.70(1.33, 2.17)^a^	1.60(1.22, 2.10)^a^
Histology				
adenocarcinoma	Ref.	Ref.	Ref.	Ref.
squamous carcinoma	1.20(1.12, 1.30)^a^	0.95(0.84, 1.08)	1.06(0.95, 1.18)	1.09(0.92, 1.30)
Others	1.16(1.05, 1.29)^a^	1.16(0.98, 1.36)	0.98(0.86, 1.12)	1.21(1.03, 1.43)^b^
Radiotherapy				
Yes	Ref.	Ref.	Ref.	Ref.
No	0.47(0.41, 0.53)^a^	0.79(0.69, 0.90)^a^	1.00(0.91, 1.10)	0.75(0.66, 0.86)^a^

a: *P*<0.01; b: *P*<0.05.

**Table 5:** Subgroup analyses stratified by TNM stage for NSCLC patients with surgery (CSS)

Characteristics	Stage I	Stage II	Stage III	Stage IV
Marital status				
Married	Ref.	Ref.	Ref.	Ref.
Divorced/separated	1.24(1.10, 1.40)^a^	1.04(0.86, 1.26)	1.13(0.98, 1.31)	1.11(0.92, 1.35)
Widowed	1.27(1.13, 1.42)^a^	1.15(0.94, 1.40)	1.21(1.04, 1.42)^b^	1.31(1.06, 1.62)^b^
Never married	1.14(0.99, 1.32)	1.26(1.04, 1.53)^b^	1.23(1.05, 1.43)^b^	0.95(0.77, 1.18)
Gender				
Male	Ref.	Ref.	Ref.	Ref.
Female	0.70(0.64, 0.77)^a^	0.83(0.72, 0.94)^a^	0.80(0.72, 0.88)^a^	0.79(0.69, 0.90)^a^
Age				
<60	Ref.	Ref.	Ref.	Ref.
60-69	1.34(1.18, 1.52)^a^	1.09(0.92, 1.29)	0.99(0.87, 1.12)	1.07(0.91, 1.26)
70-79	1.75(1.54, 1.99)^a^	1.55(1.31, 1.85)^a^	1.37(1.20, 1.57)^a^	1.22(1.01, 1.47)^b^
≥80	2.48(2.12, 2.91)^a^	1.86(1.45, 2.38)^a^	1.78(1.46, 2.17)^a^	1.48(1.14, 1.92)^a^
Race				
White	Ref.	Ref.	Ref.	Ref.
Black	1.10(0.95, 1.28)	0.83(0.66, 1.04)	0.93(0.79, 1.11)	1.10(0.89, 1.35)
Others	0.88(0.75, 1.03)	0.68(0.53, 0.86)^a^	0.93(0.78, 1.11)	0.91(0.71, 1.18)
Diagnosis year				
2004-2008	Ref.	Ref.	Ref.	Ref.
2009-2012	0.89(0.81, 0.98)^b^	0.98(0.86, 1.13)	0.86(0.78, 0.96)^a^	0.89(0.78, 1.02)
Median household income				
Quartile 4	Ref.	Ref.	Ref.	Ref.
Quartile 3	1.10(0.97, 1.24)	1.08(0.89, 1.30)	1.13(0.97, 1.31)	1.03(0.85, 1.24)
Quartile 2	1.12(0.99, 1.25)	0.89(0.75, 1.07)	1.02(0.89, 1.18)	1.02(0.85, 1.22)
Quartile 1	0.98(0.87, 1.11)	0.93(0.78, 1.12)	0.96(0.84, 1.11)	0.92(0.75, 1.13)
Grade				
I	Ref.	Ref.	Ref.	Ref.
II	1.79(1.55, 2.06)^a^	1.14(0.84, 1.55)	1.83(1.43, 2.35)^a^	1.28(0.97, 1.70)
III	2.11(1.82, 2.46)^a^	1.35(0.99, 1.83)	2.16(1.69, 2.77)^a^	1.67(1.27, 2.21)^a^
IV	1.97(1.47, 2.64)^a^	1.24(0.79, 1.95)	2.75(1.91, 3.95)^a^	1.72(1.12, 2.63)^b^
Unknown	1.60(1.31, 1.95)^a^	1.41(0.97, 2.04)	1.90(1.45, 2.51)^a^	1.74(1.30, 2.33)^a^
Histology				
adenocarcinoma	Ref.	Ref.	Ref.	Ref.
squamous carcinoma	1.04(0.94, 1.15)	0.83(0.71, 0.96)^b^	1.01(0.90, 1.14)	1.08(0.90, 1.30)
Others	1.13(0.99, 1.28)	1.18(0.99, 1.41)	0.99(0.86, 1.14)	1.22(1.03, 1.45)^b^
Radiotherapy				
Yes	Ref.	Ref.	Ref.	Ref.
No	0.39(0.33, 0.45)^a^	0.75(0.64, 0.87)^a^	0.96(0.87, 1.06)	0.72(0.63, 0.83)^a^

a: *P*<0.01; b: *P*<0.05.

### Subgroup analyses of patients without surgery stratified by TNM stage

**Figure 5:**
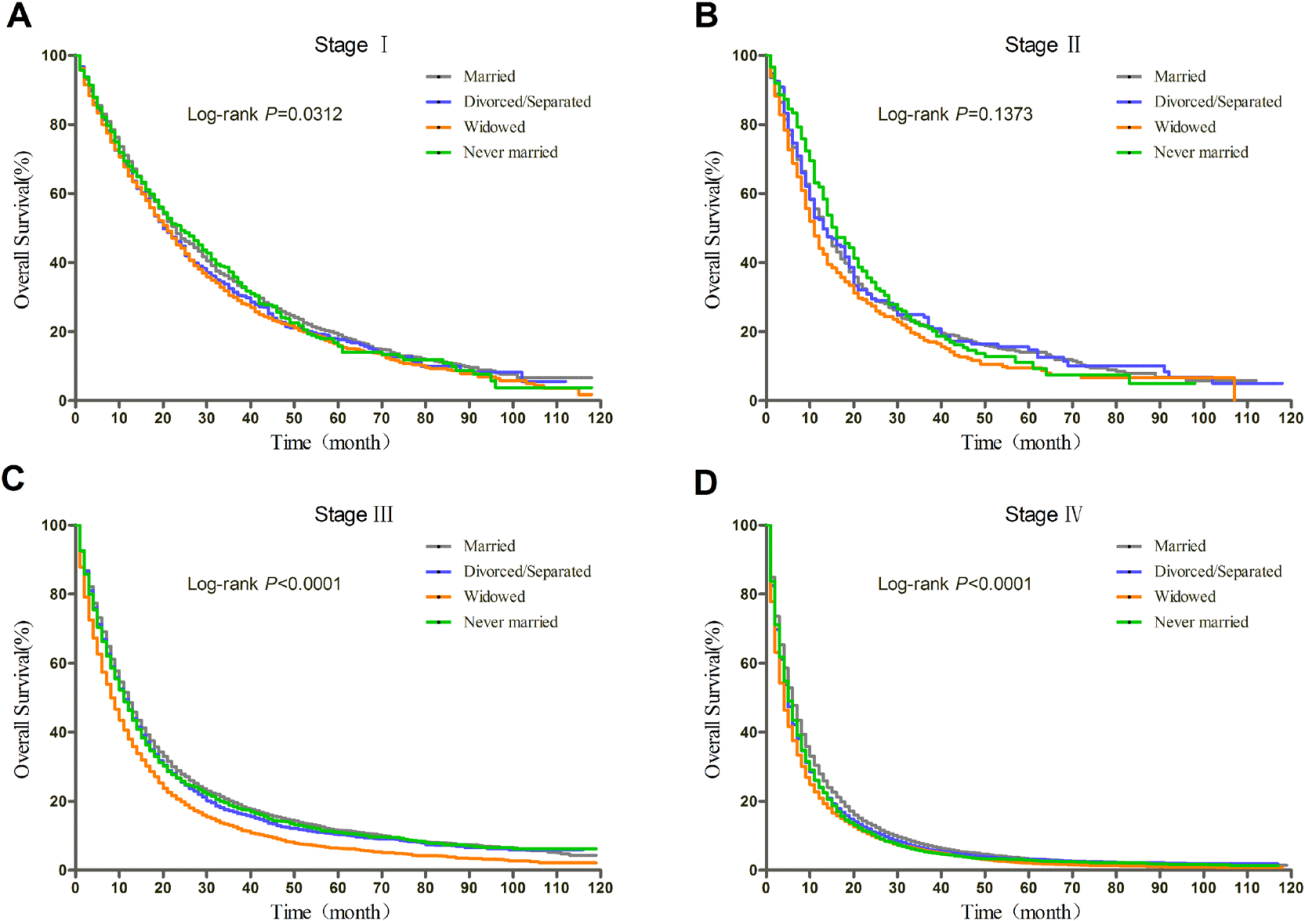
Kaplan-Meier curves of the effect of marital status on OS for patients without surgery by stage

**Figure 6:**
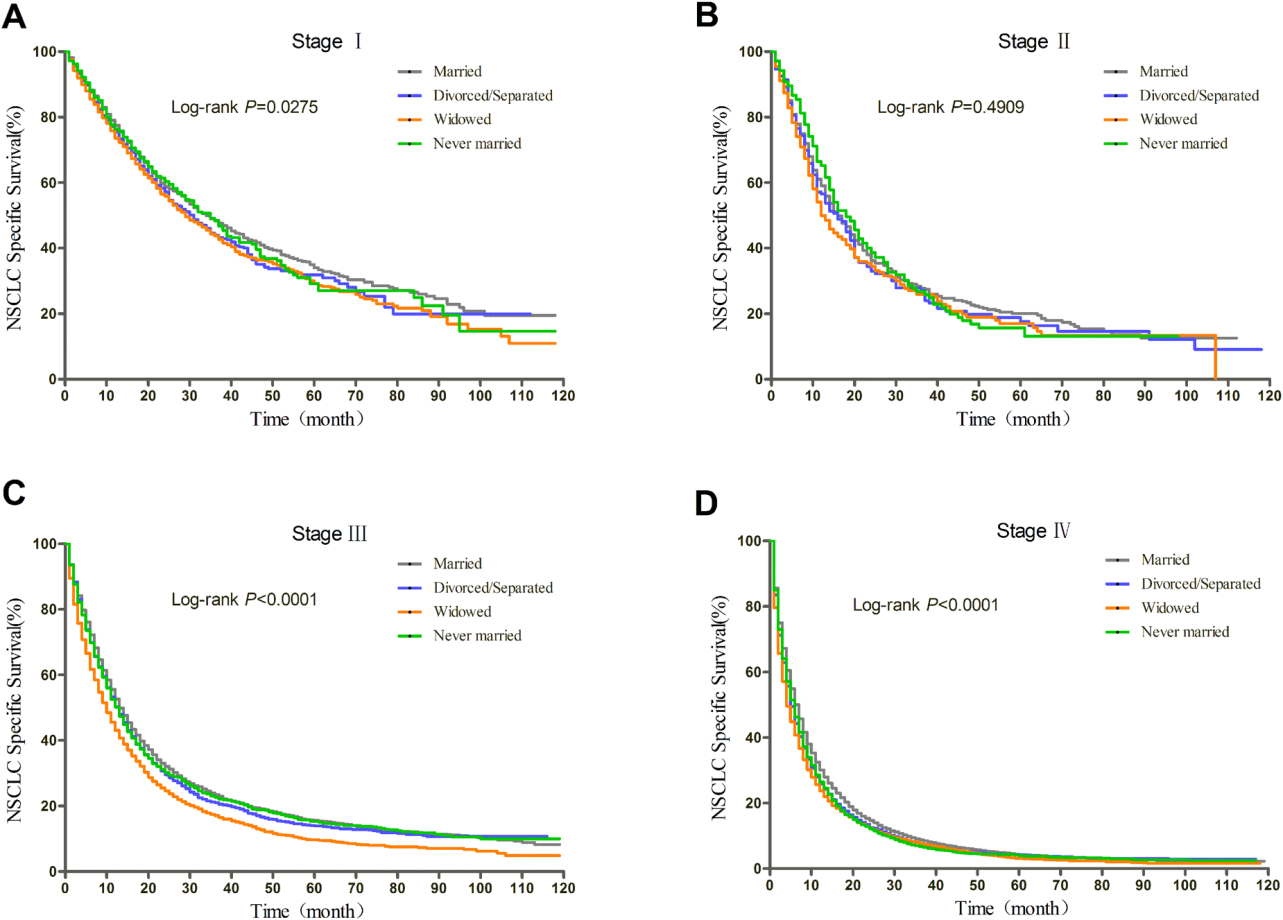
Kaplan-Meier curves of the effect of marital status on CSS for patients without surgery by stage

We also explored the effect of marital status on OS and CSS, stratified by TNM, in patients who did not receive surgery. The log-rank tests of the OS and CSS differences among different marital status were shown in Figure 5 and Figure 6, respectively. The results were summarized in Table 6 for OS and Table 7 for CSS. For patients at Stage I, divorced/separated and widowed patients had greater risk of mortality than married patients. For patients at Stage III and Stage IV, all three unmarried groups had poorer OS and CSS than married group. For patients at Stage II, however, the marital status had no relationship with the OS and CSS. Age over 60 was independent prognostic factor at all stages for OS and CSS. Contrary to the findings for patients who received surgery, not receive radiotherapy was found to be a risk factor for both OS and CSS for patients who did not receive surgery. Besides, the relationship between histology type and the OS and CSS of patients without surgery seemed to be stronger than that of patients with surgery since squamous carcinoma and other histology types were identified as independent risk factors at all stages.

**Table 6:** Subgroup analyses stratified by TNM stage for NSCLC patients without surgery (OS)

Characteristics	Stage I	Stage II	Stage III	Stage IV
Marital status				
Married	Ref.	Ref.	Ref.	Ref.
Divorced/separated	1.20(1.08, 1.34)^a^	1.03(0.86, 1.24)	1.14(1.08, 1.21)^a^	1.13(1.09, 1.17)^a^
Widowed	1.10(1.01, 1.20)^b^	1.14(0.97, 1.34)	1.17(1.12, 1.23)^a^	1.13(1.10, 1.17)^a^
Never married	1.02(0.91, 1.14)	1.04(0.85, 1.26)	1.13(1.07, 1.20)^a^	1.15(1.11, 1.19)^a^
Gender				
Male	Ref.	Ref.	Ref.	Ref.
Female	0.80(0.75, 0.86)^a^	0.82(0.72, 0.94)^a^	0.83(0.80, 0.86)^a^	0.84(0.82, 0.86)^a^
Age				
<60	Ref.	Ref.	Ref.	Ref.
60-69	1.20(1.03, 1.39)^b^	1.28(1.03, 1.60)^b^	1.13(1.07, 1.20)^a^	1.12(1.09, 1.16)^a^
70-79	1.42(1.23, 1.64)^a^	1.43(1.16, 1.76)^a^	1.31(1.24, 1.38)^a^	1.26(1.22, 1.31)^a^
≥80	1.63(1.41, 1.89)^a^	1.84(1.47, 2.29)^a^	1.65(1.56, 1.76)^a^	1.49(1.43, 1.55)^a^
Race				
White	Ref.	Ref.	Ref.	Ref.
Black	1.02(0.91, 1.13)	1.06(0.87, 1.28)	0.99(0.94, 1.05)	1.01(0.97, 1.04)
Others	0.95(0.83, 1.09)	1.06(0.83, 1.35)	0.88(0.83, 0.94)^a^	0.85(0.81, 0.88)^a^
Diagnosis year				
2004-2008	Ref.	Ref.	Ref.	Ref.
2009-2012	0.86(0.80, 0.92)^a^	0.96(0.84, 1.09)	0.93(0.90, 0.96)^a^	0.95(0.93, 0.97)^a^
Median household income				
Quartile 4	Ref.	Ref.	Ref.	Ref.
Quartile 3	0.97(0.88, 1.07)	1.28(1.06, 1.54)^b^	1.05(0.99, 1.10)	1.02(0.99, 1.06)
Quartile 2	0.91(0.82, 1.01)	1.13(0.93, 1.38)	1.08(1.03, 1.14)^a^	1.04(1.01, 1.08)^b^
Quartile 1	0.98(0.89, 1.08)	1.26(1.04, 1.53)^b^	1.03(0.98, 1.09)	0.99(0.95, 1.02)
Grade				
I	Ref.	Ref.	Ref.	Ref.
II	1.36(1.16, 1.61)^a^	1.40(0.93, 2.10)	1.19(1.04, 1.35)^a^	1.26(1.15, 1.38)^a^
III	1.39(1.18, 1.63)^a^	1.51(1.01, 2.24)^b^	1.23(1.09, 1.39)^a^	1.51(1.39, 1.64)^a^
IV	1.34(0.96, 1.87)	1.47(0.84, 2.57)	1.32(1.11, 1.58)^a^	1.61(1.43, 1.82)^a^
Unknown	1.20(1.03, 1.40)^b^	1.43(0.97, 2.11)	1.19(1.05, 1.34)^a^	1.45(1.34, 1.58)^a^
Histology				
adenocarcinoma	Ref.	Ref.	Ref.	Ref.
squamous carcinoma	1.41(1.29, 1.54)^a^	1.33(1.13, 1.56)^a^	1.20(1.14, 1.25)^a^	1.10(1.06, 1.14)^a^
Others	1.26(1.15, 1.37)^a^	1.27(1.07, 1.50)^a^	1.15(1.10, 1.20)^a^	1.17(1.14, 1.20)^a^
Radiotherapy				
Yes	Ref.	Ref.	Ref.	Ref.
No	1.90(1.77, 2.03)^a^	1.74(1.52, 1.98)^a^	1.69(1.63, 1.75)^a^	0.99(0.97, 1.01)

a: *P*<0.01; b: *P*<0.05.

**Table 7:** Subgroup analyses stratified by TNM stage for NSCLC patients without surgery (CSS)

Characteristics	Stage I	Stage II	Stage III	Stage IV
Marital status				
Married	Ref.	Ref.	Ref.	Ref.
Divorced/separated	1.21(1.07, 1.37)^a^	1.11(0.91, 1.35)	1.13(1.07, 1.20)^a^	1.13(1.09, 1.17)^a^
Widowed	1.14(1.03, 1.27)^b^	1.09(0.91, 1.31)	1.17(1.11, 1.23)^a^	1.12(1.08, 1.17)^a^
Never married	1.03(0.89, 1.18)	1.09(0.89, 1.35)	1.11(1.04, 1.18)^a^	1.13(1.09, 1.17)^a^
Gender				
Male	Ref.	Ref.	Ref.	Ref.
Female	0.81(0.75, 0.89)^a^	0.89(0.77, 1.03)	0.83(0.80, 0.87)^a^	0.85(0.83, 0.87)^a^
Age				
<60	Ref.	Ref.	Ref.	Ref.
60-69	1.20(1.00, 1.44)	1.31(1.03, 1.66)^b^	1.08(1.02, 1.14)^a^	1.11(1.07, 1.14)^a^
70-79	1.37(1.15, 1.63)^a^	1.36(1.09, 1.71)^a^	1.22(1.15, 1.29)^a^	1.22(1.18, 1.26)^a^
≥80	1.50(1.26, 1.79)^a^	1.80(1.42, 2.30)^a^	1.51(1.41, 1.60)^a^	1.41(1.35, 1.47)^a^
Race				
White	Ref.	Ref.	Ref.	Ref.
Black	0.96(0.85, 1.09)	1.03(0.83, 1.27)	1.00(0.94, 1.05)	0.98(0.95, 1.02)
Others	0.93(0.79, 1.09)	1.10(0.84, 1.44)	0.88(0.82, 0.94)^a^	0.82(0.79, 0.86)^a^
Diagnosis year				
2004-2008	Ref.	Ref.	Ref.	Ref.
2009-2012	0.81(0.74, 0.88)^a^	0.90(0.78, 1.03)	0.93(0.90, 0.97)^a^	0.95(0.92, 0.97)^a^
Median household income				
Quartile 4	Ref.	Ref.	Ref.	Ref.
Quartile 3	1.00(0.89, 1.13)	1.32(1.07, 1.61)^a^	1.04(0.98, 1.10)	1.02(0.98, 1.06)
Quartile 2	0.91(0.81, 1.03)	1.13(0.91, 1.40)	1.08(1.02, 1.14)^a^	1.04(1.01, 1.08)^b^
Quartile 1	0.93(0.82, 1.05)	1.21(0.98, 1.50)	1.04(0.99, 1.10)	0.99(0.95, 1.02)
Grade				
I	Ref.	Ref.	Ref.	Ref.
II	1.51(1.23, 1.85)^a^	1.42(0.91, 2.22)	1.23(1.07, 1.41)^a^	1.29(1.17, 1.41)^a^
III	1.51(1.24, 1.85)^a^	1.50(0.97, 2.32)	1.30(1.14, 1.49)^a^	1.54(1.41, 1.69)^a^
IV	1.36(0.90, 2.06)	1.54(0.84, 2.83)	1.45(1.20, 1.74)^a^	1.65(1.46, 1.87)^a^
Unknown	1.24(1.02, 1.50)^b^	1.41(0.92, 2.16)	1.24(1.09, 1.41)^a^	1.48(1.35, 1.61)^a^
Histology				
adenocarcinoma	Ref.	Ref.	Ref.	Ref.
squamous carcinoma	1.49(1.34, 1.66)^a^	1.33(1.11, 1.58)^a^	1.18(1.12, 1.23)^a^	1.09(1.05, 1.13)^a^
Others	1.22(1.10, 1.36)^a^	1.23(1.02, 1.48)^b^	1.09(1.04, 1.14)^a^	1.15(1.12, 1.18)^a^
Radiotherapy				
Yes	Ref.	Ref.	Ref.	Ref.
No	2.01(1.85, 2.18)^a^	1.63(1.41, 1.88)^a^	1.67(1.60, 1.74)^a^	0.97(0.95, 0.99)^b^

a: *P*<0.01; b: *P*<0.05.

## DISCUSSION

In this study, we found that unmarried patients, including divorced/separated, widowed and never married, are at significantly greater risk of poorer prognosis. After adjusting for demographics, grade, histology, stage, and treatment, marriage is still associated with a reduction in the risk of death. When we analyzed the effect of marital status on OS and CSS according to stage and surgery, the results differed on different conditions. Specifically, widowed patients who underwent surgery had poorer OS at all stages, compared with other marital status. Unmarried patients who were at Stage III and Stage IV and did not receive surgery had poorer OS and CSS, compared with married patients at the same stages.

The main findings of this study are consistent with the findings of previous observational studies conducted on other types of cancer [12, 19–21], that unmarried patients had survival disadvantage over married patients. However, the significance of this study is that we analyzed the effect of marital status on the OS and CSS according to different stages and different surgery conditions and further found that non-surgical unmarried patients had even worse prognosis when diagnosed at advanced stages and that surgical widowed patients had the worst survival at all stages, which had never been investigated for NSCLC before. Moreover, the study did not verify the conclusion drawn from several relative studies that married patients are more likely to be diagnosed with early stage, in the population of NSCLC patients, but we did find that married patients are more likely to receive surgery, which had also been demonstrated in studies.

Many explanations exist for the vital question of why unmarried status is associated with poorer overall survival and cancer-specific survival after adjustment for demographics, stage, histology, and treatment. One of the most likely reason is that unmarried patients had worse adherence to the prescribed treatments than married patients [22]. Many researches had demonstrated that adherence improves the outcome of cancer patients. For example, Li et al. found that noncompliance translated into a significant increase in the failure rate of breast-conservation therapy [23]; Mccowan et al. examined tamoxifen adherence and its relationship to mortality in women with breast cancer and concluded women who have a low adherence to tamoxifen are at increased risk of death [24]; and in head/neck cancers, delayed or missed radiotherapy is related to increased risk of locoregional recurrence and death [25].

The benefit of marriage is more than that. Psychologically, marriage can relieve a patient of the depression and anxiety caused by cancer, for a spouse can share the emotional burden and provide strong social support [26]. Depression and anxiety, in part, may be a mediator of the association between marital status and the adherence of treatment, and between adherence of treatments and outcomes. Studies have demonstrated a strong relationship between marriage and the depression degree, and that the depression has a negative relationship with the adherence to treatment [27, 28]. Besides, a spouse may motivate a patient’s desire to live so the patient is more likely to take the courage to receive certain progressive therapy such as surgery. Physiologically, marriage has been found to be beneficial for endocrine, cardiovascular, and the function of immune system, though the effect magnitude primarily depends on the quality of marriage [29]. Moreover, adequate social support may lower the level of cortisol, which have been linked with natural-killer cell count and survival in cancer patients [30–32], providing a hypothesis of the basis mechanism explaining why marriage has survival advantages over ones who are not married.

The SEER database provides us the opportunity to perform large, population-based studies. However, there are several limitations that should be addressed and the results of the study should be interpreted with caution. An obvious limitation of the SEER database is the relative lack of control variables beyond simple demographics and clinical factors. For example, the database is unable to provide important confounding variables such as smoking habit, comorbidity of patients, and other treatment procedures. Secondly, marital status is not a static entity, which means marriage dissolve and reform with the progress of time. This is particularly true for elderly NSCLC patients who may have been “married” at the time of diagnosis, but “widowed” at the time of death. Thirdly, “married” may not necessarily mean “harmony” and “support”. The quality of marriage and the satisfaction degree that patients get from the marriage also have key importance on the outcome. But the database does not contain such information. Finally, this study is a retrospective observational study, so it has all the inherent limitations that an observational study may have.

Despite these potential limitations, our study indicates that unmarried patients including divorced/separated, widowed, and never married, are at greater risk of overall death and NSCLC cause-specific death. The relationship seems stronger for widowed patients and patients at advanced stages. When caring for the unmarried cancer patients, doctors should make more efforts to emphasize to patients the importance of following the advice of doctors and pay more attention to abnormity of susceptible systems during the treatment. Health care systems should take measurements to provide social support for this population to minimize the risk of death caused by marital status.

## MATERIALS AND METHODS

### Data source

The data utilized in this study was obtained from SEER program, which consists of 18 registries covering approximately 28 percent of the US people and routinely collects information of cancer patients including demographics, primary tumor site, cancer stage, treatment and the follow up information for survival. The database is an authoritative source of information on the incidence and survival of cancer in the United States and it has been used for many studies on the research of prognostic factors associated with various cancers [33–36].

### Inclusion criteria

Patients with ICD-O-3 (International Classification of Diseases for Oncology, 3^rd^ edition) site code C34.0-C34.9 from 2004 to 2012 was extracted from the SEER database. The inclusion criteria were as follows: (a) ICD-O-3 morphology code did not indicates small cell lung cancer (8041, 8042, 8043, 8044, and 8045);(b) age at diagnosis was older than 18 years; (c) marital status was known; (d) diagnosed with NSCLC only or multiple primary cancers but NSCLC was the first; (e) known survival time and survival time was greater than 0 month; (f) known cause of death;(g) definite AJCC TNM stage (h) known surgery and radiotherapy information.

### Study variables and outcomes

Study variables in this study included gender, age at diagnosis, race, year of diagnosis, tumor grade, histologic type, AJCC TNM stage, surgical information and radiotherapy information. The patients were divided into four groups according to age (<60, 60-69,70-79, and ≥80). Race was grouped by white, black, and others (including American Indian/Alaska native, Asian/Pacific Islanders, etc.). Year of diagnosis was divided into 2004-2008 and 2009-2012. Histologic type was classified as adenocarcinoma, squamous carcinoma and others. Tumor grade I-IV represented well differentiated, moderately differentiated, poorly differentiated, and undifferentiated, respectively. Surgery and radiation were both categorized as yes (received) and no (did not receive). The “no” of the variable Surgery represented no surgery of primary site or autopsy only; while the “yes” included local tumor destruction or excision, excision or resection of less than one lobe, lobe or bilobectomy, lobe or bilobectomy extended, pneumonectomy, and extended pneumonectomy. We also included county-level median household income using the Census American Community Survey data to represent patients’ socioeconomic status. The variable was divided into quartiles: Quartile 1(<US $51250), Quartile 2(US $51250-62230), Quartile 3(US $62231-71180), and Quartile 4(>US $71180). Marital status was classified as married, divorced or separated, widowed, and never married (including single, unmarried or domestic partner).

The primary outcomes were overall survival (OS) and NSCLC cause-specific survival (CSS). OS was calculated as the number of month from diagnosis to death due to any cause. CSS was calculated as the number of month from diagnosis to death due to NSCLC. Patients who died from other causes or were still alive at the end of the study period were defined as censored.

### Statistical analyses

The baseline characteristics of patients with different marital status were summarized and compared using chi-square test. Kaplan-Meier log-rank test was adopted to compare the difference of OS and CSS between subgroups of each variable. Multivariate Cox analysis was conducted to compare the OS and CSS of patients with different marital status after adjusting various covariates. We further conducted subgroup analyses, stratified by different AJCC TNM stage and whether the patient received surgery, to assess the risk of marital status and other variables on OS and CSS more specifically. All P values were two-sided, and the values less than 0.05 were considered statistically significant. All analyses were performed using SAS version 9.4 (SAS Institute Inc., Cary, NC, USA).

## Abbreviations

NSCLC: Non-small cell lung cancer
OS: Overall survival
CSS: Cause-specific survival
AJCC: American Joint Committee on Cancer
